# Evaluation of color perception in cataract patients bilateral implanted with presbyopia-correcting intraocular lenses

**DOI:** 10.1038/s41598-025-18259-5

**Published:** 2025-10-03

**Authors:** Haokun Qu, Yubing Huang, Yun Chen, Weitao Zheng, Zheming Wu, Lina wu, Zheng Wang, Ruihong Ju

**Affiliations:** 1 Foshan Aier ZhuoYue Eye Hospital, Foshan, 528000 Guangdong China; 2 Aier Eye Hospital, Jinan University, Guangzhou, 510071 Guangdong China

**Keywords:** Cataract, Intraocular lenses, Presbyopia, Color perception, FM-100 hue test, Lens diseases, Vision disorders

## Abstract

To evaluates postoperative color perception in cataract patients implanted with various yellow-tinted intraocular lenses (Y-IOL) and clear intraocular lenses (C-IOL), including extended depth of focus (EDOF) and trifocal lenses, using the Farnsworth-Munsell 100 Hue test. An observational clinical study was conducted on patients undergoing cataract surgery with bilateral implantation of either Y-IOL or C-IOL at Guangzhou Aier Eye Hospital. Patients were categorized into Y-IOL and C-IOL groups based on lens tint and further subcategorized by design (EDOF vs. trifocal, Y-trifocal vs. C-trifocal). The FM-100 Hue test assessed total error score root mean square (TES RMS), angle, C-Index, S-Index, and total test duration three months postoperatively. Statistical analyses included Kruskal-Wallis, ANOVA, and Mann-Whitney U tests. Among 54 patients (108 eyes), no significant differences in TES, angle, C-Index, S-Index, or test duration were observed between Y-IOL and C-IOL groups or between EDOF and trifocal IOL groups (all *P* > 0.05). However, a significant difference in the S-Index was noted between the Y-trifocal and C-trifocal IOL subgroups (*P* < 0.05), indicating differing color axis scatter tendencies. The implantation of blue-light filtering IOLs does not significantly affect color perception when compared to clear IOLs. The design differences between presbyopia-correcting IOLs, such as extended depth of focus and trifocal IOLs, also had minimal impact on patients’ postoperative color vision, except for a notable difference in scatter index between two trifocal IOLs.

## Introduction

Cataracts, characterized by lens opacity or discoloration, are one of the most common ocular conditions, ranking as the second leading cause of visual impairment and the primary cause of blindness globally^1,2^. The most effective treatment for cataracts involves removing the opacified lens through phacoemulsification (PHACO), followed by the implantation of an intraocular lens (IOL) to restore visual function. With continuous advancements in surgical techniques and the growing variety of IOL options, cataract surgery has evolved beyond its traditional therapeutic role, now commonly regarded as a refractive procedure aimed at optimizing visual outcomes^3,4^.

With the continuous advancement of technology, the design of intraocular lenses (IOLs) has become increasingly sophisticated. Notably, the development of blue light filtering intraocular lenses (BFIOLs) has introduced a novel option for cataract surgery, providing enhanced visual outcomes and potential protective benefits against blue light exposure. BFIOL through specific designs, effectively filter short-wave blue light (wavelength ranging from 400 to 500 nm), thereby potentially reducing damage to the retina especially macular and improving visual quality for patients^5–8^. This type of IOL is also known as yellow-tinted IOL (Y-IOL) because of its yellow color appearance. However, the application of blue-light filtering has also sparked extensive discussions about its impact on color perception^9–12^. Color perception is an integral part of the human visual system which begins with the absorption of light by three types of cone photoreceptors in the retina, leading to a series of neural transformations that ultimately result in color perception^13,14^.

Research indicates that cataract patients frequently encounter issues such as reduced color saturation and decreased contrast^15^, which not only affect their quality of life but may also have negative impacts on their mental health^16,17^. Therefore, investigating the specific effects of different types of IOLs on color vision is expected to drive the development of more personalized cataract treatment plans, thereby offering the potential for further enhancement of postoperative visual quality. Existing studies predominantly focus on the impact of monofocal Y-IOL on color vision, yet the effects of functionally enhanced Y-IOLs such as extended depth of focus IOL (EDOF IOL) and trifocal IOL on postoperative color perception remain unclear. Thus, the aim of this study is to determine color perception following implantation of difference types of IOLs using Farnsworth-Munsell 100 (FM-100) Hue tests.

## Methods

### Ethical approval

Written informed consent was gained by all participants. According to the Helsinki Declaration, the study was approved by the ethics committee of Aier Eye Hospital, Jinan University (Guangzhou, China; No.GZAIERIRB2020003).

### Study designs and patients

This observational clinical study was conducted at Guangzhou Aier Eye Hospital. It involved patients who underwent cataract surgery and were implanted with the same type of functionally enhanced IOL in both eyes between June 2023 to February 2024. The patients were divided into two groups based on the different IOLs: yellow-tinted IOL group (Y-IOL group) and the clear IOL group (C-IOL group). Similarly, patients were categorized based on different intraocular lens designs, such as extended depth of focus IOL group (EDOF IOL group) and trifocal IOL group. The EDOF IOL group was further divided into Y-EDOF and C-EDOF subgroups based on whether the lens filters blue light. The trifocal IOL group was further divided into Y-trifocal group (TFNT) and C-trifocal group (tri839).

The implanted IOL in Y-IOL group included AcrySof IQ Vivity (Alcon Laboratories, Inc.), AcrySof IQ PanOptix TFNT (Alcon Laboratories, Inc.) and TECNIS Synergy (ZFR00V) IOL (Johnson & Johnson Vision, Santa Ana, CA, USA). The implanted IOL in C-IOL group included Tecnis Symfony (ZXR00) IOL (Johnson & Johnson Vision, Santa Ana, CA, USA) and AT LISA tri 839 MP (Carl Zeiss Meditec, Jena, Germany) (Table 1 for details).

The IOLs in EDOF IOL group included AcrySof IQ Vivity, TECNIS Synergy (ZFR00V) and Tecnis Symfony (ZXR00) IOL while AcrySof IQ PanOptix TFNT and AT LISA tri 839 MP in trifocal IOL group.

The Y-EDOF IOL group included AcrySof IQ Vivity, TECNIS Synergy (ZFR00V). Tecnis Symfony (ZXR00) IOL were divided into C-EDOF IOL group (Table 1 for details).

**Table 1:** The IOLs included in different groups.

Group	Included IOLs
Y-IOL	AcrySof IQ Vivity, AcrySof IQ PanOptix TFNT, TECNIS Synergy (ZFR00V)
C-IOL	Tecnis Symfony (ZXR00), AT LISA tri 839 MP
EDOF IOL	AcrySof IQ Vivity, TECNIS Synergy (ZFR00V), Tecnis Symfony (ZXR00)
Trifocal IOL	AcrySof IQ PanOptix TFNT, AT LISA tri 839 MP
Y-EDOF IOL	AcrySof IQ Vivity, TECNIS Synergy (ZFR00V)
C-EDOF IOL	Tecnis Symfony (ZXR00)
Y-trifocal IOL	AcrySof IQ PanOptix TFNT
C-trifocal IOL	AT LISA tri 839 MP

The inclusion criteria were as follows: (1) lens opacity; (2) Expected postoperative astigmatism ≤ 1.00 D; and (3) Alpha angle < 0.5 mm and Kappa angle < 0.3 mm. Exclusion criteria are as follows: (1) severe corneal clouding that prevents passage of the laser; (2) severe systemic disease (e.g. diabetes); (3) other eye diseases such as glaucoma, age-related macular degeneration, etc.; (4) prior history of refractive surgery.

### Pre-operation examination

All patients underwent preoperative evaluation, which included slit-lamp biomicroscopy, non-contact intraocular pressure measurement, corneal specular microscopy and topography, fundus examination, as well as iTrace ray tracing aberrometer assessment. The IOLMaster 700 (Carl Zeiss Meditec, Jena, Germany) was utilized to measure axial length and calculate IOL power using the Barrett Universal II formula with a refractive target of plano. Degree of lens opacification grading was determined using dysfunctional lens index (DLI) testing conducted by iTrace (Tracey Technologies, Houston, USA).

### Main outcomes

The main outcome measures were total error score root mean square (TES RMS), Angle, C-Index, and S-Index and total test duration in FM-100 hue test within 3 months postoperatively between Y/C-IOL group, also in Y/C-EDOF IOL group and Y/C-trifocal IOL group. Each patient included in the study underwent at least one or more FM-100 hue tests within three months after bilateral cataract extraction with implantation of the same type of IOL. Each test result for each patient was considered an independent result and used for data analysis.

The Angle (confusion angle) serves to identify the type of color vision deficiency by analyzing the direction of errors in the arrangement of color samples, thereby determining the primary axis of color vision abnormality (e.g., the red-green axis or the blue-yellow axis). The C-Index (Confusion Index) quantifies the severity of color vision deficiency, reflecting the degree to which the test-taker deviates from the perfect arrangement of color samples. The S-Index (Confusion Index) assesses the “polarity” or “randomness” of the arrangement of color samples, indicating whether the test-taker’s errors are concentrated in a specific direction (e.g., one single color axis). The TES RMS is the sum of the differences in errors of adjacent positions of color samples, reflecting the overall color discrimination ability, while the C/S-index and the angle further refine the type and pattern of the deficiency^18,19^.

### Farnsworth-munsell 100 Hue test

All patients were tested under a standard light source of natural light with an incidence angle of 90° and an observation angle of 60°. Total error score root mean square (TES RMS), Angle, C-Index, S-Index and total testing duration were used to evaluate color perception. FM-100 Hue test is based on binocular vision, so we only tested patients who had cataract surgery in both eyes. Therefore, the unit of count is the number of patients.

### Cataract surgery

All surgeries were performed by four experienced surgeons (ZM.W, Y.C, HS.C, RH.J) following standardized procedures. The LenSx platform (Alcon Laboratories, Inc., Fort Worth, TX, USA) was used for capsulotomy (5 mm) and lens fragmentation (energy parameter, 10µJ). PHACO surgery was performed using the standardized phacoemulsification with a 2.4 mm tip and sleeve, utilizing the Alcon Centurion system (Alcon Laboratories, Inc., Fort Worth, TX, USA), followed by IOL implantation into the capsular bag.

### Study lenses

The AcrySof IQ Vivity IOL is the first IOL that addresses presbyopia using non-diffractive EDOF optics. The IOL is made of an hydrophobic acrylate/methacrylate copolymer with UV and blue light filters, has an optic diameter of 6 mm and an overall length of 13 mm^20^. AcrySof IQ PanOptix TFNT is a 1-piece aspheric hydrophobic presbyopia-correcting IOL which designed to have an intermediate focal point of 60 cm blue-light filtering trifocal IOL^21^. The TECNIS Synergy OptiBlue IOL, model ZFR00V, has a proprietary diffractive surface derived from a combination of extended depth-of-focus and multifocal technologies. In addition, the Synergy IOL includes violet light–filtering chromophore, which reduces transmittance of violet light wavelengths^22^. The ZXR00 is a 6.0 mm biconvex hydrophobic acrylic monofocal IOL with an aspheric anterior surface which expands the depth of focus across the principles of the echelette diffractive ring^23^. AT LISA 839 is a diffractive trifocal implant with 3.33 addition for near and 1.66 addition for intermediate, with 6 mm optic diameter and a total diameter of 11 mm^24^.

### Statistical analysis

The statistical analysis was performed using SPSS 26.0 for Windows (SPSS Inc., Chicago, Illinois, USA). Descriptive statistics were utilized to assess the data. The normality of data distribution was evaluated using the Kolmogorov-Smirnov test. Between-group differences for normally distributed variables were assessed using the Kruskal-Wallis and one-way ANOVA tests, while non-normally distributed variables and qualitative information were analyzed using the Wilcoxon and Mann-Whitney U tests. For normally distributed data, continuous and categorical variables were described as mean ± standard deviation (SD) and number, respectively, with statistical significance set at *P* < 0.05.

## Results

### Baseline characteristics

Table 2 shows the baseline characteristics. 46 eyes in 23 patients were included in the C-IOL group, with a mean age of 59.83 ± 11.85 years and mean DLI of 3.25 ± 2.34. 62 eyes in 31 patients were included in the Y-IOL group, with a mean age of 59.18 ± 11.37 years and mean DLI of 3.81 ± 2.59. There were no significant differences found in age, preoperative visual acuity, axial length (AL), DLI, and average K.

**Table 2:** Baseline characteristics.

	C-IOL	Y-IOL	*P* Value
Eye No. (%)	46 (42.6)	62 (57.4)	/
Patients No. (%)	23 (42.6)	31 (57.4)	/
Age (years)	59.83 ± 11.85	59.18 ± 11.37	0.7212
Male	10	15	/
Female	13	16	/
Axial Length (mm)	26.35 ± 2.43	25.40 ± 2.10	0.0524
Visual Acuity (LogMar)	1.04 ± 0.61	0.99 ± 0.54	0.6808
DLI	3.25 ± 2.43	3.81 ± 2.59	0.2179
IOL Power (D)	13.73 ± 6.94	15.99 ± 5.54	0.0727
Average K (D)	43.09 ± 11.49	42.89 ± 1.62	0.6366

### Color perception

**Fig. 1:**
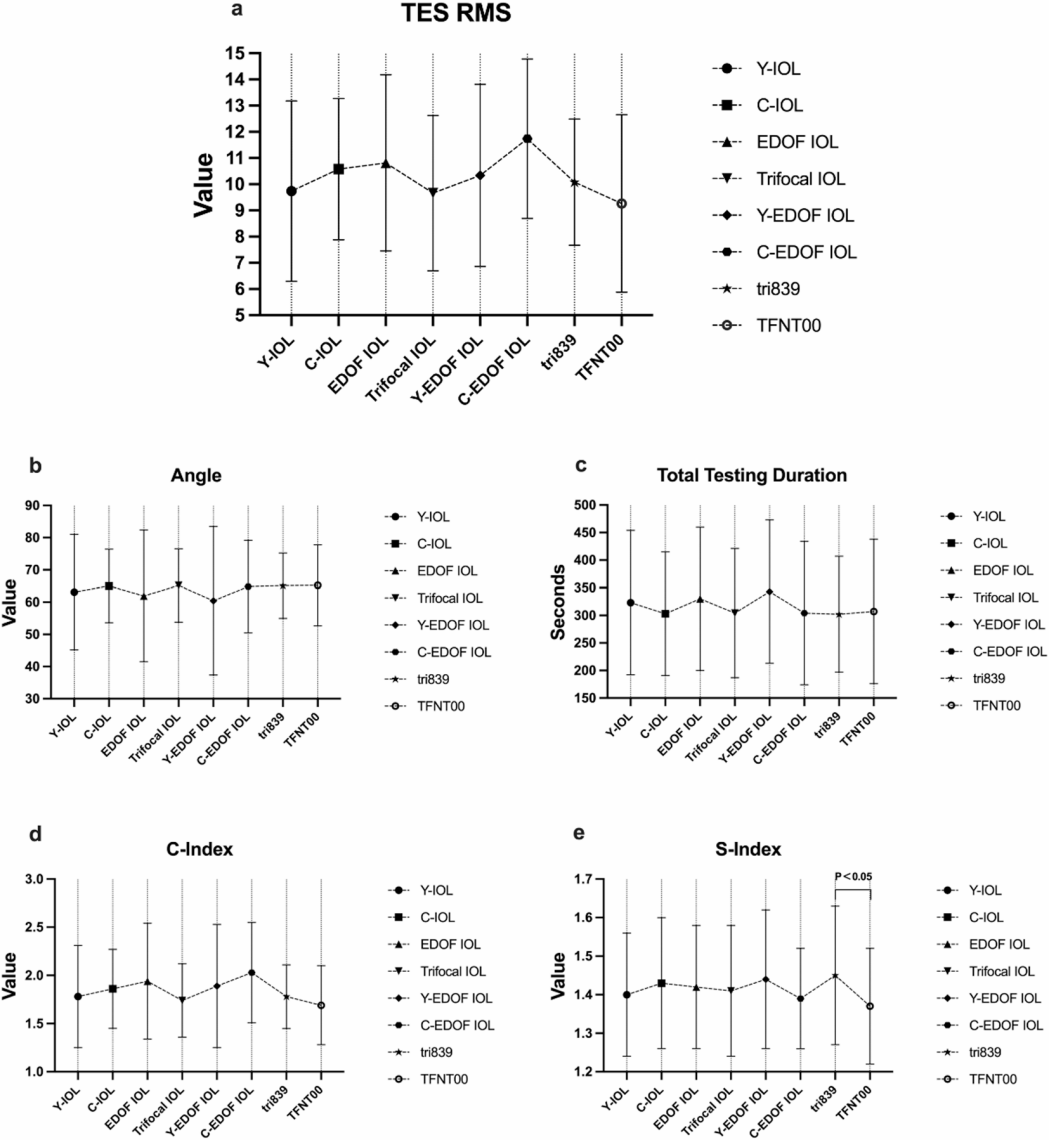
Results of TES RMS (**a**), Angle (**b**), total testing duration (**c**), C-Index (**d**) and S-Index **(e**) between all the groups.

Table 3; Fig. 1 show the result of TES RMS, C-Index, S-Index and total testing duration between C-IOL group and Y-IOL group within 3 months postoperatively. No significant difference was found in TES RMS, Angle, C-Index, S-Index and total testing duration between 2 groups.

**Table 3:** Results of TES RMS, C-Index, S-Index and total testing duration between C-IOL group and Y-IOL group within 3 months postoperatively.

	C-IOL	Y-IOL	*P*-Value
Total Patients-times	50	66	/
TES RMS	10.576 ± 2.694	9.735 ± 3.441	0.0999
Angle	65.03 ± 11.41	63.11 ± 17.94	0.9578
C-Index	1.86 ± 0.41	1.78 ± 0.53	0.1006
S-Index	1.43 ± 0.17	1.40 ± 0.16	0.3531
Total Testing Duration (S)	303 ± 112	323 ± 131	0.4979

Table 4; Fig. 1 show the results of FM-100 Hue test results between EDOF IOL group, trifocal IOL group, Y-EDOF group and C-EDOF group within 3 months postoperatively. No significant difference was found in TES RMS, Angle, C-Index, S-Index and total testing duration between these groups.

**Table 4:** Results of TES RMS, C-Index, S-Index and total testing duration between EDOF IOL group, trifocal IOL group, Y-EDOF group and C-EDOF group within 3 months postoperatively.

	EDOF IOL	Trifocal IOL	Y-EDOF	C-EDOF
Total Patients-times	44	72	29	15
TES RMS	10.814 ± 3.364	9.660 ± 2.961	10.336 ± 3.474	11.738 ± 3.038
Angle	61.93 ± 20.43	65.17 ± 11.36	60.43 ± 23.05	64.82 ± 14.35
C-Index	1.94 ± 0.60	1.74 ± 0.38	1.89 ± 0.64	2.03 ± 0.52
S-Index	1.42 ± 0.16	1.41 ± 0.17	1.44 ± 0.18	1.39 ± 0.13
Total Testing Duration (S)	330 ± 130	304 ± 117	343 ± 130	304 ± 130

Table 5; Fig. 1 show the results of FM-100 Hue test results Y-trifocal group and C-trifocal group within 3 months postoperatively. A significant difference was found in S-Index between 2 groups (*P* < 0.05).

**Table 5:** Results of TES RMS, C-Index, S-Index and total testing duration between Y-trifocal group and C-trifocal group within 3 months postoperatively.

	Y-trifocal	C-trifocal	*P*-Value
Total Patients-times	37	35	/
TES RMS	9.263 ± 3.388	10.078 ± 2.410	0.2791
Angle	65.21 ± 12.55	65.11 ± 10.13	0.8658
C-Index	1.69 ± 0.41	1.78 ± 0.33	0.2069
S-Index	1.37 ± 0.15	1.45 ± 0.18	0.0442*
Total Testing Duration (S)	307 ± 131	302 ± 105	1

*P < 0.05.

## Discussion

Whether blue-light filtering IOL alters patients’ color perception has been the subject of much debate in the past. Chromatic aberration arises when light of different wavelengths is refracted unevenly as it passes through a lens, causing each color to focus at distinct points. This phenomenon leads to color fringing and image distortion, which can significantly degrade optical quality^25,26^. Intraocular lenses are often designed with regions of varying light transmission characteristics. For instance, in certain designs, the central area can transmit nearly 100% of the light, while the peripheral regions significantly reduce the amount of light transmission^27^.

Early studies suggested that its ability to filter short wave length blue light leads to deficits in color vision perception, especially in blue color discrimination^28,29^. However, recent studies have shown that there is no difference in color perception between yellow-tinted IOLs and traditional clear IOLs after implantation^12^.

However, the above conclusions are primarily discussed based on studies involving monofocal IOLs. Whether the combination of different optical designs of functional intraocular lenses with blue light filtering affects patients’ color perception due to variations in chromatic aberration remains a topic worthy of further investigation. To our knowledge, there are currently no studies exploring the clinical outcomes of color vision perception in patients after implantation of blue-light filtering trifocal or EDOF IOLs. Therefore, this study conducted the FM-100 color vision test on patients, focusing solely on those who had cataract surgery on both eyes and received the same type of IOL among cataract patients.

At the present stage, the quantification of differences in the appearance color of IOLs can be achieved through the CIELAB color space. Developed by the International Commission on Illumination (CIE), the CIELAB color space aims to create a color quantification system that aligns more closely with human visual perception^30^. This system measures the reflectance spectrum of an object’s surface using a spectrophotometer and then converts it into CIELAB coordinates along three orthogonal axes: lightness, red-green axis, and yellow-blue axis. Color difference (ΔE) is calculated based on the corresponding coordinates on three orthogonal axes^31^. However, existing research reports indicate a significant lack of application of the CIELAB system to different types of IOLs. The manner and proportion in which different IOLs distribute light energy to multiple focal points may vary, potentially affecting ΔE. Therefore, a classification discussion of IOLs with different optical designs is still necessary. Additionally, there is a lack of specific ΔE values for different lenses, and subgroup analyses are not conducted for IOLs with varying degrees of yellowness.

This study included data from both eyes of each patient, despite the potential influence of the correlation between test results of both eyes on statistical outcomes^32^, which is disadvantageous for discussing the effect of a single type of IOL on patients’ color vision perception. However, color vision is inherently a binocular cooperative process, and the test results reflect the integrated effect of bilateral inputs. Given that the FM-100 Color Vision Test requires participants to integrate color information using both eyes, the scores reflect overall color vision rather than independent performance of each eye. Therefore, this study treats the test results from both eyes of each patient as a single independent observation unit, which aligns with actual clinical application scenarios.

Our research findings indicate that there were no significant differences in TES RMS, Angle, C-index, S-index, and total test time between the Y-IOL group and the C-IOL group or in EDOF IOL group. This suggests that bilateral implantation of two different types of intraocular lenses does not affect patients’ color perception except different trifocal IOLs. This conclusion is consistent with the results of monofocal IOL studies. To protect the retina from potential photodamage, numerous IOL products have been engineered as yellow lenses^33^. This is achieved by covalently bonding or physically blending a yellow chromophore capable of absorbing blue light within the polymeric material. This additive selectively absorbs the blue portion of the spectrum while permitting the transmission of other wavelengths (such as green, yellow, and red light), thereby imparting a pale-yellow appearance to the IOL^34^. Due to the more complex design of multifocal IOLs compared to monofocal IOLs, there are more factors at the lens design level that can influence patients’ postoperative color perception, particularly with trifocal IOLs. This may also explain the statistically significant difference in the S-Index between the Y-trifocal and C-trifocal group observed in this study. The S-Index in Y-trifocal group is significantly lower than C-trifocal group. It implies that in the Y-trifocal group, errors in color axis alignment are more concentrated compared to the C-trifocal group, indicating a stronger tendency towards color misguidance. Although there was a statistical difference in the S-Index between the two groups in this study, more research is needed to investigate whether this reflects a clinically significant difference in follow-up.

Whether more sophisticated and complex optically based IOLs will emerge in the future, and whether these IOLs will in turn have a more pronounced effect on color vision with the addition of blue light filtering, is still a question worth exploring.

Clear IOLs block only ultraviolet light, whereas the natural lens gradually yellows with age and naturally filters some of the blue light (400–500 nm). When a clear IOL replaces the natural lens, the transmission of blue light increases, and this mechanism may lead to postoperative cyanosis^35^. However, the results of the present study do not seem to support this conclusion. The reasons for these results will be explained in the following paragraphs of this paper.

The impact of blue-light filtering IOL implantation on patients’ color vision depends on lighting conditions. The light source used in this study was natural light, which falls under the category of photopic conditions. This is one of the potential reasons why the results of our study did not exhibit significant differences. One study found that under scotopic conditions, the blue discrimination ability of eyes implanted with blue-light filtering IOLs was lower than that of eyes with clear IOLs, while under mesopic and photopic conditions, there was no difference in color recognition ability between the two^36^. A possible explanation is that the visual system contains three types of cone cells, each sensitive to long, medium, and short wavelengths of light, respectively. These three types of cone cells are influenced by a comparison mechanism, which produces the perception of color^37^. When ambient brightness decreases, the amount of light projected onto the macular region of the retina is reduced, diminishing the comparison mechanism and resulting in differences in color perception. Studies have shown that under low light conditions, human color perception can change, potentially causing discrepancies between perceived and actual colors. For example, a chromaticity that appears pink under higher brightness may appear yellow under lower brightness levels^38^.

Age factor also affects the patient’s color perception. The long-wavelength light (red and yellow) receptors in the human eye are more susceptible to aging, and compared to young people, the elderly are more prone to errors in color vision tests^39^. Equally, however, with age, the human visual system exhibits certain adaptive and compensatory mechanisms in processing color information. One study examining the effects of macular degeneration on color vision showed that suprathreshold parafoveal color perception remained largely unaffected, despite the fact that selective absorption of short-wavelength light by the retina increases with age, resulting in decreased color sensitivity^40^. This indicates that the visual system can to some extent compensate for changes in color perception caused by variations in refractive media, which may also account for the lack of difference in color perception ability between the two IOLs in this test. From the results of this study and recent reports on similar research, it appears that for professions with high color cognition requirements, such as painters and photographers, the implantation of yellow IOLs or clear IOLs does not affect postoperative color recognition abilities.

Our study aims to explore the impact of different IOLs implanted after cataract surgery on color vision. At this time, it is unknown how many “yellowness” an IOL will or will not affect a patient’s postoperative color perception. At least in this study, differences in the interference color judgment outcomes have been observed among trifocal IOLs from different manufacturers. It is foreseeable that the debate over whether the implantation of a Y-IOL after cataract surgery will affect color vision will continue.

The innovation of our study is using a subjective approach to compare color perception between Y-IOLs and clear IOLs in many types of functional IOLs, and to compare the effects of color perception after IOL implantation with different focal design. However, our study still has several shortcomings need to be improved. The first is that we have not collected the results of color vision tests from patients preoperatively. This is because the purpose of this study was to clarify the effect of IOL implantation on color vision rather than to explore the effect of cataract surgery per se on color vision, and the effect of color changes occurring in the aging lens on color perception needed to be excluded. The second is that the results of this study include multiple test outcomes from the same patient. It remains inconclusive whether repeated testing in the FM-100 hue test enhances proficiency and thereby influences the results. Thirdly, the follow-up period of this study was only three months, which is a short period of time that may cause errors in the postoperative data and conclusions. However, considering that color vision is a subjective feeling and the length of adaptation time is not the same for each individual, a follow-up period of three months is sufficient. Fourthly, control group data from a healthy young population were not included. It is because this study focused on physiological color vision changes in postoperative patients. Longer follow-up and larger sample sizes are still needed in the future to study the effects of different IOLs implanted on color vision perception.

The study was supported by Natural Science Foundation of Hunan Province (2024JJ9035); The Scientific Research Foundation Project of Guangzhou Aier Eye Hospital, Jinan University (GA2023004); Medical Science and Technology Research Foundation Project of Guangdong Province (C2018042). All the authors of this paper do not have any potential conflict of interest.

## Conclusion

The implantation of blue-light filtering IOLs does not significantly affect color perception when compared to clear IOLs. The design differences between presbyopia-correcting IOLs, such as extended depth of focus and trifocal IOLs, also had minimal impact on patients’ postoperative color vision, except for a notable difference in scatter index between two trifocal IOLs.

## Data Availability

The datasets generated and/or analysed during the current study are not publicly available but are available from the corresponding author, RH Ju, upon reasonable request.
